# Nurses' Looks

**Published:** 1916-07-29

**Authors:** 


*To the Editor of* The Hospital.

### Nurses' Looks.

*To the Editor of* The Hospital.

Sir,—The remark is often heard that nurses as a body are a very plain set of women, but is not this apparent lack of ordinary good looks really due to the way in which the uniform is put on, and also to the way in which so many nurses do their hair? Why, because a woman wears a cap, should she strain her hair off her face in the effort to be tidy? Some nurses go to the other extreme and do their hair in too elaborate a fashion. Probationers usually have to be shown how to put on their caps. Might they not also have it impressed upon them that great care should be taken to do the hair tidily but at the same time becomingly?—Yours faithfully,

July 18, 1916. A Matron.

